# How do critical care staff respond to organisational challenge? A qualitative exploration into personality types and cognitive processing in critical care

**DOI:** 10.1371/journal.pone.0226800

**Published:** 2020-01-08

**Authors:** K. E. Grailey, E. J. Murray, J. Billings, S. J. Brett

**Affiliations:** 1 Department of Surgery and Cancer, Imperial College London, London, United Kingdom; 2 Saïd Business School, University of Oxford, Oxford, United Kingdom; 3 University College London, London, United Kingdom; Middlesex University, UNITED KINGDOM

## Abstract

Critical care staff are frequently required to respond to stressful scenarios. The way staff counter organisational challenge may be influenced by their underlying personality type, preferred style of cognitive processing and previous clinical experience. Our objective was to explore the personality types of a sample of critical care workers, and the potential relationship of this with cognitive processing. This was achieved through a qualitative interview study in which participants were presented with difficult but realistic scenarios pertaining to staffing. Data on individual’s personality were captured using the ‘16 Personality Factor’ assessment, a tool that produces scores for 16 different elements of an individual’s personality. The existence of perfectionist and pragmatic cognitive processing styles were identified as one theme emerging from a prior analysis of these interview transcripts. We aimed to validate this, explore our ability to categorise individuals into groups based upon their cognitive processing. We identified that some individuals strongly tended to either a perfectionist or pragmatic style of cognitive processing for the majority of their decisions; however most adapted their style of processing according to the nature of the decision. Overall participants generally demonstrated average scores for all 16 personality factors tested. However, we observed that some factors tended to higher scores than others, indicating a pattern within the personalities of our sample cohort. Whilst a small sample size, our data suggests that individuals working within the same critical care environment may have clear differences in their approach to problem solving as a consequence of both their personality type and preferred style of cognitive processing. Thus there may be individuals within this environment who would benefit from increased support to minimise their risk of cognitive dissonance and stress in times of challenge.

## Introduction

The Intensive Care Unit (ICU) can be a stressful working environment. The pressure associated with the management of complex clinical situations, bed and staff shortages and the requirement to respond to deteriorating patients from within the rest of the hospital and beyond can be intense for those responsible for the outcome. [1,2]

A previous qualitative study [3] by this team explored staff (nurses, doctors and physiotherapists) perceptions of clinical risks and safe staffing levels within the ICU. The study aimed to identify and increase understanding about the cognitive processes exhibited by staff members.

Qualitative interviews were comprised of two components. Staff were initially asked to discuss a fictitious staffing scenario, designed to provide a “level of organisational challenge that might normally be encountered” [3]. As part of the scenario, participants were required to allocate staff to each bed space, but would not be able to achieve ideal staffing ratios. As such this scenario utilised clinical knowledge and organisational strategy, whilst also providing a point from which further discussion could develop. The second component took the format of questions regarding risk and safety within the ICU environment. An emergent theme was the presence of differences in an individual’s ideals when conducting their work. Some individuals demonstrated preferences based upon a “perfectionist” model whereas others were “pragmatic” in nature. The analysis indicated that when striving for perfection for an individual patient, staff members might have an increased tendency to suffer cognitive dissonance—distress due to experiencing contradictory mental pressures. This appeared to be in contrast to more pragmatic individuals who were aware that compromises needed to be made to deliver the best achievable care across a service in spite of organisational challenges.

It is well established that individuals have differing personality characteristics, which can be explored using validated personality tests such as the Sixteen Personality Factor Analysis (16PF) [4]. 16PF is a personality assessment tool based upon the personality sphere concept, and Cattell’s Handbook (1974ed) outlines typical profiles for a number of different professions, including general practitioners and nurses [5]. However, the predominance of different personality traits in ICU workers, and whether this influences working behaviour is not well explored.

16PF has been previously used within the healthcare setting to evaluate the traits of those who perform well, either in their clinical career [6] or at medical school [7]. It has also been used to explore factors influencing job satisfaction and its potential usefulness in aiding recruitment. [8] The assessment and interpretation of personality dimensions is complex, and one of many environmental and contextual factors which can influence an individual’s behaviour. Nevertheless, 16PF has been used in several industries to successfully identify specific personality traits in their workforce, which can be contrasted against the typical trait distribution seen in the general population.

In addition to being interviewed, participants completed 16PF personality assessments, the results of which were not explored in the original paper. The objectives of this analysis were to interrogate the 16PF data and build on the findings of the previous study to further explore cognitive processing in critical care workers.

As such our specific aims were to:

1. Explore the 16PF personality assessments of our sample of critical care workers, with a view to identifying the presence of any significant trends or predominant traits. Through this we aimed to evaluate whether common personality traits of critical care workers can be described or predicted.
2. Categorise participants into groups based upon their preferred style of cognitive processing and investigate the presence of any associations between an individual’s style of cognitive processing and their dominant personality traits.

## Materials and methods

Data were collected in the original study from three adult ICU’s within one hospital group between November 2014 and 2015. Written informed consent was obtained from participants with provision of written materials prior to commencement of the qualitative interviews. Data consisted of anonymised transcribed audio transcripts of the qualitative interviews with ICU staff. The original topic guide for these interviews can be viewed in S1 File.

These were matched with participant’s anonymised 16PF personality assessments using identifiers. As per U.K research governance regulations, research studies involving staff members do not require ethical committee review, but we obtained review and approval from Imperial College Healthcare NHS Trust’s and Imperial College’s Joint Research Compliance Office (Reference number 13HH1823).

16PF Personality assessment data for each participant were compared and assessed for predominance of certain traits. During the assessment participants complete 185 multiple choice questions. The raw scores for each of the 16 personality factors are converted to Standard Ten scores (or STEN) and it is these scores which are provided in the personality report for each candidate [9]. These scores represent a point on a continuum where an individual’s personality (for each trait being measured) sits in relation to the personality of the wider population, indicating how an individual may behave naturally (as opposed to learnt behaviour). It does not represent an actual score itself for that trait. When providing assessment results to participants, their position on the scale is referred to as either “left handed” with scores ≤ 3 (also defined as a low score) or “right handed”–with scores ≥ 8 (also referred to as high scores). Scores 4–7 are described as average. Distribution and dominance of each participant’s 16PF scores were analysed using Excel (Microsoft, Redmond, Washington, USA).

Corresponding interview transcripts were re-coded applying a thematic analysis technique utilising computer assisted qualitative analysis software package NVIVO (QSR International, Victoria, Australia) in order to evaluate participants’ predominant response to organisational challenge. The data were initially sorted to identify any cognitive processing as described by the individual, (descriptions of decisions/actions made by others were excluded from this analysis). These were then evaluated for pragmatic or perfectionist tendencies (identified as a theme in the previous study) and coded accordingly. This was performed by a researcher (KG) who was aware of this theme, but had not reviewed prior detailed coding or data analysis. Statements were reviewed independently by a member of the original coding team (EM) with subsequent discussions within the research team to assess for inter-rater agreement in interpretation.

A content analysis technique was utilised, and participants were categorised according to whether the majority of statements they made describing their response to organisational challenge were perfectionist, pragmatic, or a balance between the two. Individuals were allocated to either the perfectionist or pragmatic group if most of their statements were consistent with one type of response.

The two sets of data were triangulated and subsequently analysed using Prism (V7, GraphPad, La Jolla, California USA) to explore the presence of any association between an individual’s response to organisational challenge and their behavioural tendencies as outlined by their 16PF personality profile. The distribution of 16PF traits within each group (“perfectionist”, “pragmatic” or “balanced”) was re-evaluated to see if there were any differences in the predominant personality traits.

## Results

Thirty-one participants completed a 16PF personality assessment in addition to participating in the qualitative interview. Participant characteristics are described in Table 1.

**Table 1:** Participant characteristics by gender and seniority.

Participant Characteristics	Total	Nurse	Physician	Physiotherapist
N (%)	31	17	11	3
**Gender**				
Male	10	3	5	2
Female	21	14	6	1
**Seniority**				
Senior Grade	13	6	5	2
Middle Grade	6	6	0	0
Junior Grade	12	5	6	1

**Fig 1:**
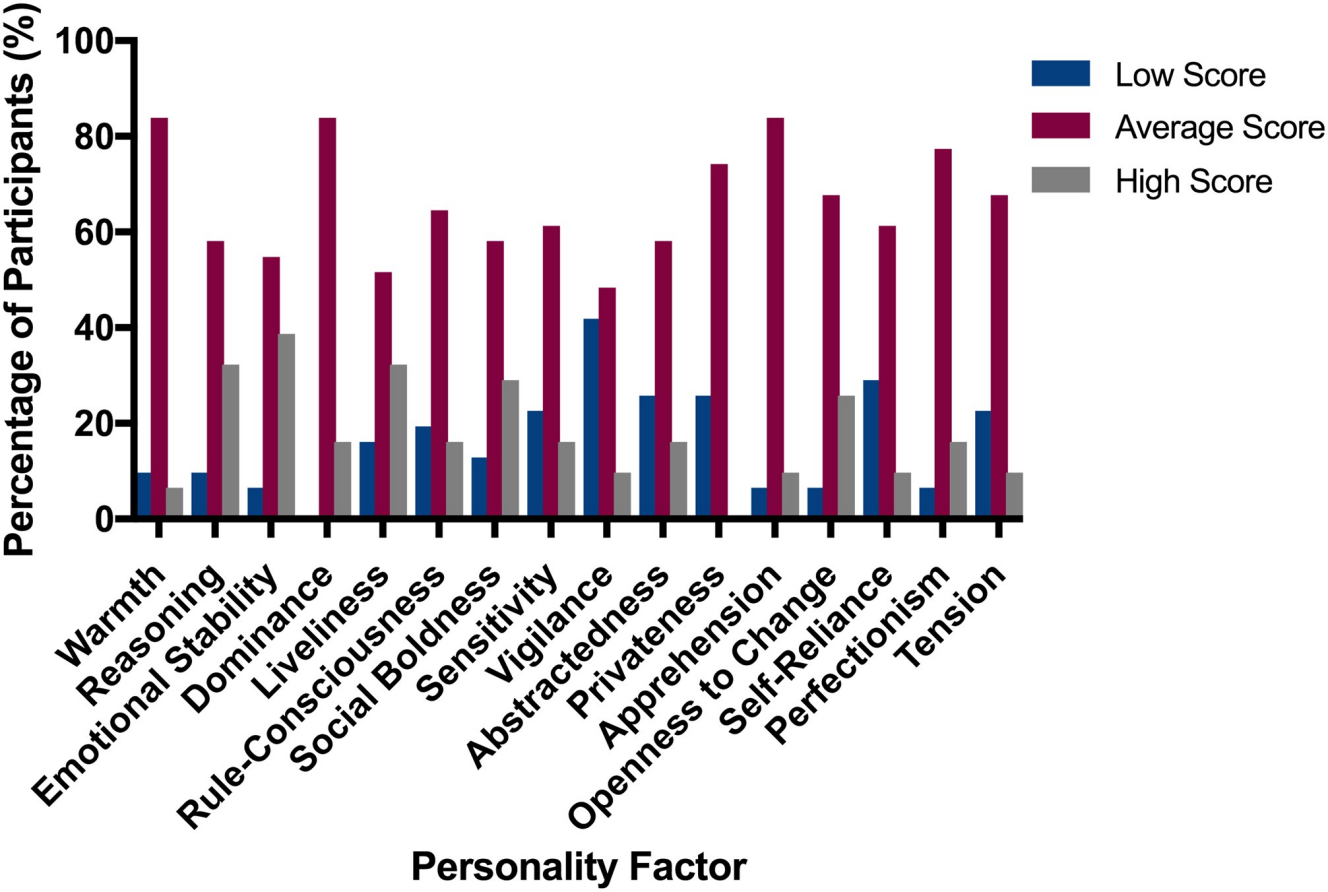
Prevalence of personality factors in all participants. (n = 31).

Most individuals in the study sample demonstrated “average” scores for all 16 personality factors. The proportions of participants scoring low, average or high scores for each factor is presented in Fig 1. Some factors demonstrated less variability within the scores than others–in warmth, apprehension and dominance few individuals scored either high or low values. No individuals scored highly for privateness or were low scoring for dominance. Notably, a large proportion of participants demonstrated low scores for vigilance. The definitions, and behaviours that compromise each personality factor are provided in S1 Table.

Of note, Cattell’s handbook refers to each Factor with a letter (A-Q_4_), with descriptors for extremes of each factor [5]. Cattell’s original academic psychology terminology has been translated into “lay” terms for each factor in later versions of the 16PF. The results of any analysis using the 16PF needs to be interpreted with caution, and the different definitions of each factor making up the 16 components of personality need to be reviewed prior to interpretation.

One thousand three hundred and fifty-five statements within the qualitative interview transcripts described behaviours or cognitive processing that arose in response to a challenge presented by the interviewer. Of these, 277 statements corresponded with either a pragmatic or perfectionist approach and were coded as such: one hundred and sixty-six statements were perfectionist in nature and 111 were pragmatic. Discussion between the research team and comparison of 10% of the coded transcripts provided confidence in the reproducibility of this coding scheme.

Examples of responses which were coded as perfectionism:

- *"We cannot transfer the patient*, *we have to make sure that this patient has got no pain before we transfer*, *because at least the patient is here in ITU*, *we can closely monitor them*, *so just for example*, *the patient is not ready to go in the ward*, *during our shift."*
- *"You absolutely have to prioritise my patient*. *You have to send me the nurses*. *I need to wash and roll*. *Literally*. *I can’t be any clearer"*

Examples of responses which were coded as pragmatic:

- *"you do the best with what you’ve got and don’t whinge because it’s not going to help anything*…….*But I do think the more experienced you are*, *the more philosophical*."
- *"you just do what you can do and prioritise*, *and then just pick up as the slack goes off as best you can*"

An overview of these themes and subthemes with additional supporting quotations is displayed in S2 Table.

Nineteen individuals demonstrated a balance between the two styles of response, with the variation appearing to be related to the nature of the clinical challenge they faced. Twelve individuals demonstrated a strong tendency for a particular style of responding to such challenges: 4 pragmatists and 8 perfectionists.

**Fig 2:**
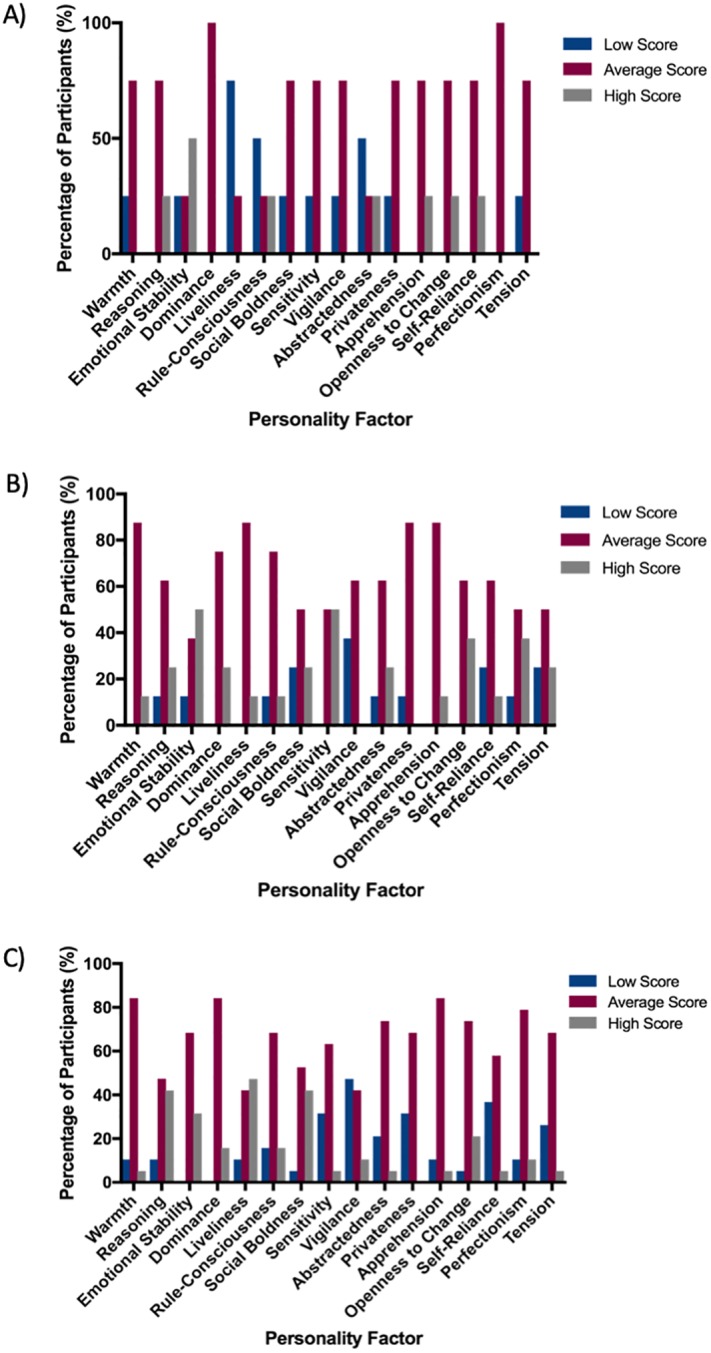
**A. Personality Profiles of Participants with a Predominantly Pragmatic Tendency.** (n = 4). **B. Personality Profile of Participants with a Predominantly Perfectionist Tendency**. (n = 8). **C. Personality Profile of Participants with a balance between Perfectionist and Pragmatic decision making.** (n = 19).

16PF profiles were subsequently divided into three groups according to the participants’ response to organisational challenge (identified within the qualitative data). Whilst acknowledging these groups are small; differences in the distribution of high and low scores for each of the 16 personality factors begin to emerge. Individuals in the pragmatic group had a predominance of low scores for liveliness, rule-consciousness and abstractedness, and high scores for emotional stability. The perfectionist group also demonstrated high scores for emotional stability, but with no factor predominating in the low range. Individuals who were identified as more balanced in their responses appeared to show a slight predominance of high scores for liveliness [Fig 2A, 2B and 2C].

## Discussion

Our data indicate that the personality of critical care workers may demonstrate a common, though not universal pattern. Most participants demonstrated mid-range scores for each personality factor. The interpretation of mid-range scores is explained in the 16PF Comprehensive Insights report [10] “A person’s specific behaviour is the product of the interaction of their personality characteristics with specific situational opportunities and constraints”. This interaction effect is most likely to be present for 16PF scores in the midrange on the profile. This may explain why the majority of individuals were able to adapt their responses depending upon the specific question posed by the clinical scenario. Notably, a large proportion of participants demonstrated low scores for “vigilance”. Vigilance is described by Cattel as the balance between “trust and suspicion”. Higher scores for vigilance are associated with scepticism, distrust and opposition, whilst lower scores are associated with being trusting and accepting. This may explain why lower scores predominated here. Reasoning and liveliness both had high scores for a third of participants, consistent with individuals who are intelligent, fast learners, abstract thinkers and enthusiastic, qualities all desirable in critical care workers.

Typical profiles for medical professionals (namely general practitioners, nursing staff and psychiatric technicians) can be found in Cattell’s original 16PF handbook. Of note, he states that it is “remarkable” that the physicians’ personality factors map closely to that of the general population; with the exception of high reasoning, low dominance, high self-reliance, strongly developed perfectionism and low tension. Cattell notes: “Apparently general practitioners are, personality-wise, almost as varied as the general public whom they serve, for the above differences, though significant, are not large”.

In contrast, nursing staff are presented as having a different personality profile, with high reasoning, high emotional stability, high perfectionism and high sensitivity.

Our analysis does not correlate entirely with these descriptions, as we did not demonstrate a strong signal for perfectionism or low tension. However, our study population is a mixed sample of healthcare workers, smaller and from a different clinical area to the medical professionals sampled. It is likely that the clinical situation and external stressors faced by critical care workers today is very different from those in general practice / general nursing at the time the 16PF handbook was written. Our study supports the argument that there is more work to be done in understanding the personality factors of critical care workers, especially taking into account differences in job stressors, education and staffing levels that have occurred since Cattell’s original work. Our exploratory data demonstrated a signal that different factors predominate in our sample in comparison to other professions, and possibly other specialties within the healthcare profession. Further exploration of these differences and the potential implications for individual stress in the clinical environment may be beneficial.

Our thematic analysis further supports the pragmatic and perfectionist theme that originated from the initial constant comparative analysis [3]. It is apparent from both qualitative analyses that individuals apply their cognitive processing in a number of different ways when faced with identical clinical scenarios and organisational challenges, and that competing perfectionist and pragmatic tendencies are present in many and one or other may dominate in some.

Perfectionism is a multi-dimensional characteristic, summarised as a ‘person striving for flawlessness and setting high performance standards’ [11, 12]. It may be accompanied by critical self-evaluation and concerns regarding others’ actions. This can be positive, but also mal-adaptive (due to the potential consequences of setting such demanding standards and striving for them despite a huge personal cost) [13]. In contrast, pragmatic individuals deal with scenarios flexibly and realistically based upon practical rather than theoretical considerations [14]. Pragmatists tend to have the aim of finding realistic solutions to problems and are more willing to compromise to achieve a desired outcome.

We were able to classify individuals into groups according to their predominant responses to organisational challenge. This exploratory study supports the idea that different behavioural groups exist within the ICU environment. Some participants were strongly perfectionist or pragmatic, although the majority were able to adapt their behaviours according to the specific task in hand. It is possible that individuals who reside in the middle ground may be at risk of being pushed into one particular style of decision making outside their comfort zone as a consequence of increased organisational stress. Conceivably, the resilience of these individuals may be positively (or negatively) impacted by leadership and team dynamics. Reviewing the statements by predominantly perfectionist individuals, increased stress appeared to be more detrimental to those with a tendency to perfectionism, leading to a narrowed focus on the immediate task in hand. This appeared to correspond with a retreat from wider situational awareness, and possibly an increased risk of cognitive dissonance–as individuals expressed concern about not being able to manage the clinical situation according to their perfectionist ideals. The main trigger for this type of response was difficulty in managing inadequate resources, with an associated avoidance of acknowledging the needs of the unit and wider organisation.

Analysis of the qualitative interview transcripts of the pragmatic individuals indicated that they appeared to be less distressed when having to make compromises, in comparison to the more perfectionist individuals who struggled with decisions that did not align with their perfectionist ideals.

Triangulation of the data through the utilisation of qualitative interview data and quantitative 16PF survey results provided further opportunity for the exploration and understanding of cognitive processing within the critical care unit. Did this analysis support the idea that personality type influenced an individual’s cognitive processing and the way in which they responded to challenges? The numbers within each group are too small to be able to draw robust conclusions, but the composition of individual personalities did differ between these groups, with some trends emerging. Those with strong preferences for *either* pragmatic or perfectionist responses had high scores for emotional stability when compared to the balanced group. Interestingly, (whilst acknowledging that this mapping of 16PF personality profiles onto these types of behaviours is weak), the predominance of certain personality factors did not correlate with those we might have predicted based upon published 16PF descriptors [S1 Table]. For example, we had expected that the perfectionist group would score highly for rule-consciousness and perfectionism, and the pragmatic group to score highly for reasoning. Again, the 16PF trait definitions do not necessarily correspond to the commonly understood dictionary definitions, but instead are a reflection of a number of personality qualities distributed along a spectrum. For example, a high score for reasoning does not mean “thinking in a logical or sensible way”, as would be defined by the Oxford Dictionary [15], but instead individuals who are abstract, fast learners and bright, as opposed to ‘concrete’ thinkers with lower mental capacity [16, 17]

This study has several limitations. The relatively small sample size, whilst providing a large amount of qualitative data, when divided into groups according to response to challenge only allows for the inference of trends rather than robust associations. We acknowledge that the secondary coding of the interview transcripts emerged from the findings of the previous study, and as such the original methodology and semi-structured interview topic guides were not designed with this specific thematic and content analysis in mind. We feel confident however that the dataset offers a narrative discussing issues related to the primary research, but that was not explored fully in the initial analysis. We mitigated the potential loss of researcher reflexivity through regular discussions between the new coder (KG) and the rest of the research team.

It may also be prudent to consider that the environment in which the individuals completed the qualitative interview was different to where they completed the 16PF tool. It is possible that completion of the 16PF tool in an environment which is less associated with stress (e.g. at home) in comparison to being presented with a stressful clinical scenario (even though fictitious) within an individual’s place of work, may have an impact upon the concordance of these two data sets. This raises a number of questions–does personality profiling and the use of tools such as 16PF have a role in our population of critical care workers, given the chaotic and unpredictable nature of their working environment? Does a tool that examines personality utilised in a controlled setting adequately predict how an individual will behave when under pressure?

Our data suggest that organisations may need to provide increased support and responsive leadership for those with perfectionist tendencies due to their increased risk of cognitive dissonance and potential for stress and isolation over time. In addition, those who have a more balanced style of processing, may need similar support and leadership to prevent them being pushed by circumstances into one extreme style of behaviour that may be detrimental to both themselves and the efficiency of the team. It is likely that multiple influences lead to the outcome of individuals demonstrating different styles of cognitive processing. These include an individual’s underlying personality, team dynamics, the decision itself, available resources, individual stress and cognitive bias based upon similar previous experiences. It may also be of benefit to review the response of individuals to different organisational challenges, again with the aim of identifying those which are modifiable. Perhaps the aim should not be to try and change individuals’ behaviour or select for job roles based upon it, but to understand, adapt and provide an environment with high psychological safety for all team members, as all individuals have a valuable input. This requires sensitive and informed leadership.

## Conclusion

Whilst the sample size within our study is modest, it provides a clear indication that individuals respond to the same stressors in different ways. It also suggests a relationship between personality type and method of cognitive processing that could be detrimental to the individual if they are not adequately supported by their team and the organisation’s leadership.

Further qualitative and mixed methods studies could be developed to evaluate further the impact of different responses to challenge and personality traits on individuals and teamworking. In healthcare it is not possible to perform the same level of matching personality to role as in other industries. However, understanding these relationships may allow us to manage our teams better, delivering more reliable care for patients in a manner which is less detrimental to professionals, perhaps reducing the incidence of stress and early burnout and preserving the most experienced part of our workforce.

## Supporting information

S1 File Qualitative interview topic guide. Staff Perceptions of Risk and Safety Qualitative Study Interview Protocol.
(PDF)

S2 File Original data—Perfectionist vs pragmatic and original 16PF scores. (XLSX)

S3 File Original data—Anonymised 16PF scores. (XLSX)

S1 Table High and low descriptors for each of the 16PF personality traits. (DOCX)

S2 Table Thematic analysis of responses to organisational challenge. (DOCX)
