# Supplementary material for: How do critical care staff respond to organisational challenge? A qualitative exploration into personality types and cognitive processing in critical care
Source: PLoS One. 2020 Jan 8;15(1):e0226800. doi: 10.1371/journal.pone.0226800 (PMC6948735; doi:10.1371/journal.pone.0226800)
Supplement: S1 File — Staff Perceptions of Risk and Safety Qualitative Study Interview Protocol. (PDF) [file pone.0226800.s001.pdf]

Imperial College Healthcare NHS
NHS Trust

S1 File : Qualitative Interview Topic Guide

Staff Perceptions of Risk and Safety Qualitative Study

Protocol

1. Brief the participant on the purpose of the project and interview.
2. Describe the nature of the interview (i.e. that it is recorded) and discuss confidentiality.
3. Describe the format of the questions. The interview consists of one decision making scenario, with a series of prompts for exploring decision-making.
4. Explain the process for completing the personality questionnaire [either after this interview or at another time [email will be sent with details of how to complete it].

Staffing Scenario

Prompts [show the participant the intensive care unit scenario, including the staffing table and ask:]

- Please can you allocate the staffing to patients for this shift?
- What additional information would you want to know?

In this type of scenario:

- How many nurses/therapists/admin/medics do **you** think are required to run this shift safely?
- What are your three key priorities for the shift?
- With the current staffing, do you have any safety concerns?
    - If yes, what are they?
    - If no, why not?
- What are the barriers in providing safe patient care?
- What are you thinking about?
- What makes you feel comfortable or challenged?
- Is there anything that this has made you think about, that we haven't covered?

Imperial College Healthcare NHS
NHS Trust

Intensive Care Unit Scenario

BED 3
Bariatric.
Long-term weaning.

BED 4
Ventilated. NIV plus filter with inotropes.

Intubated, ventilated, community-acquired pneumonia. Weaning.
SIDEROOM 2

C.difficile. Two day post-op. WHIPPLES. Waiting on ward.
SIDEROOM 3

BED 5
Community-acquired pneumonia. Weaning

BED 6
On filter. Noradrenaline. Weaning. Low-dose inotropes

BED 2
Level 3 patient to become Level 2. Agitated, restless, confused.

Intensive Care Unit Scenario

You have started a shift on a 12 bedded intensive care unit with four side rooms, occupied by 12 patients.

There are seven Level 3 patients and five Level 2 patients.

BED 7
Long-term weaner.
1st time off ventilation.
Tracheostomy mask trial for ventilation overnight.

BED 8
Oesophogectomy
Epidural.
Lots of pain.

BED 1
Neurological patient.
High ICPs.
Needs urgent CT scan.
Paralysed and sedated.
Family 24/7.

Handover notes

Booked admissions:
2 x electives
1 x ventilated patient, intubated on ward

Discharges:
1 x predicted
1 x confirmed (patient with epidural)

Neurosurgical patient
MRI to clear their neck.
Paralysed and sedated otherwise stable.
SIDEROOM 1

Just extubated.
Confused and agitated. Head injury.
Waiting for trauma bed.
SIDEROOM 4

Band	Nursing	Therapists	Admin	Grade	Medical
7	1	1 (off sick)		Cons	1
6	3 (1st day/1 agency)	1		Reg	1
5	6 (1 junior supervised by Band 6)	2 (both supervised)		F2/ST	2
3	1		1(ward clerk sick)		

Staffing Issues

- Can you recall shifts where a staffing problem significantly affected the quality of patient care?
- What was the staffing problem?
- How was patient care affected?
- What were the implications of the situation for you, your colleagues and the unit?

Perception of Risk

- What do you feel is a high risk staffing situation on the unit? Why?
- Is that situation viewed as high risk by your colleagues? By your team leader/manager?
- What staffing situations do you consider as too high risk?

Perceptions of Safety

- Tell me about a time when you were concerned about safety in your area?
- Did you discuss this with your team leader/manager?
- How did the conversation go?
- Did you team leader/manager act on the safety issue you described?
- Has the safety issue been addressed?
- What could be done in the department to improve patient and safety experience?
- Which factors prevent admitting patients/admissions?
