# Supplementary material for: How do critical care staff respond to organisational challenge? A qualitative exploration into personality types and cognitive processing in critical care
Source: PLoS One. 2020 Jan 8;15(1):e0226800. doi: 10.1371/journal.pone.0226800 (PMC6948735; doi:10.1371/journal.pone.0226800)
Supplement: S1 Table — (DOCX) [file pone.0226800.s004.docx]

**S1 Table: High and Low Descriptors for each of the 16PF Personality Traits**

| **16PF Scale Names and Descriptors** | | |
| --- | --- | --- |
| **Descriptors of Low Range** | **Primary Scales** | **Descriptors of High Range** |
| Reserved, Impersonal, Distant | **Warmth** | Warm-hearted, caring, attentive to others |
| Concrete, Lower mental capacity | **Reasoning** | Abstract, Bright, Fast-Learner |
| Reactive, Affected by feelings | **Emotional Stability** | Emotionally Stable, adaptive, mature |
| Deferential, Cooperative, Avoids Conflict | **Dominance** | Dominant, Forceful, Assertive |
| Serious, Restrained, Careful | **Liveliness** | Enthusiastic, Animated, Spontaneous |
| Expedient, Nonconforming | **Rule-consciousness** | Rule-Conscious, Dutiful |
| Shy, Timid, Threat-Sensitive | **Social Boldness** | Socially Bold, Venturesome, Thick-skinned |
| Tough, Objective, Sentimental | **Sensitivity** | Sensitive, Aesthetic, Tender-minded |
| Trusting, Unsuspecting, Accepting | **Vigilance** | Vigilant, Suspicious, Sceptical, Wary |
| Practical, Grounded, Down-to-Earth | **Abstractedness** | Abstracted, Imaginative, Idea-Orientated |
| Forthright, Genuine, Artless | **Privateness** | Private, Discreet, Non-disclosing |
| Self-assured, Unworried, Complacent | **Apprehension** | Apprehensive, Self-Doubting, Worried |
| Traditional, Attached to Familiar | **Openness to change** | Open to Change, Experimenting |
| Group-Orientated, Affiliative | **Self-Reliance** | Self-Reliant, Solitary, Individualistic |
| Tolerates Disorder, Unexacting, Flexible | **Perfectionism** | Perfectionistic, Organised, Self-Disciplined |
| Relaxed, Placed, Patient | **Tension** | Tense, High Energy, Driven |

Adapted by The Sage Handbook of Personality Theory and Assessment ^(14)^ with permission from S.R Conn and M.L. Rieke (1994). 16PF Fifth Edition Technical Manual ^(15)^
