# Supplementary material for: How do critical care staff respond to organisational challenge? A qualitative exploration into personality types and cognitive processing in critical care
Source: PLoS One. 2020 Jan 8;15(1):e0226800. doi: 10.1371/journal.pone.0226800 (PMC6948735; doi:10.1371/journal.pone.0226800)
Supplement: S2 Table — (DOCX) [file pone.0226800.s005.docx]

**S2 Table: Thematic analysis of responses to organisational challenge.**

| **OVERVIEW OF DECISION MAKING TENDENCY THEME AND SUBTHEMES WITH ASSOCIATED PRACTICAL EXAMPLES AND SUPPORTING QUOTATIONS.** | | | | |
| --- | --- | --- | --- | --- |
| **MAIN THEME** | **SUBTHEME BASED UPON RESPONSE TO ORGANISATIONAL CHALLENGE** | **DEFINITION OF SUBTHEME** | **EXAMPLES** | **SUPPORTING QUOTATIONS** |
| RESPONSE TO CHALLENGE | Pragmatic | Willing to compromise to achieve best possible result with limited resources.  Realistic approach to problem solving  Efficient and task driven. | Able to balance demands of external hospital with needs of unit  More flexible with implementation of policy and protocol depending upon clinical need.  Accepts less than perfect care to provide good quality care to more patients. | "in view of the real extreme short staffing there you’re not going to push your luck.  So you would actually save them to do that on another day when they could have more support and perhaps just look after one patient"  "we are encouraged to try to think ‘outside the envelope’ – I hate that expression – but we’re encouraged to think about what you actually need and what would be nice to have."  "I would be using the relatives where I could as well because nothing beats a relative holding somebody’s hand to help with the agitated patient.  So I would be looking at that sort of resource as well as just the nurses."  "you do the best with what you’ve got and don’t whinge because it’s not going to help anything.......But I do think the more experienced you are, the more philosophical."  "It would be very difficult. It may be that with this Band 5, I would also have her to help out and between the three of us do it."  “The two electives, I’d hope that by the next shift that there’s someone else who’s on and they’d be liaising with recovery that they could keep them for a little while and that they’d come in later'   "you just do what you can do and prioritise, and then just pick up as the slack goes off as best you can"  "you just pick a plan at the time and deal with whatever’s happening."  "And then I would decide on, about(?) the electives, and what indications for operation they have, if they’ve got cancer or something else life threatening, prioritise that one"  "you have to be flexible"  "So perhaps this is a workable strategy assuming everybody is staying on the ward"  "So staffing is always an issue but as with everything people just survive and get through the shift."  "I think there’s an acceptable staff rate but it’s not the optimal staff rate.  What is acceptable is quite different." |
|  | Perfectionist | Constant striving for high performance and best possible outcome  Self-critical and critical of others with respect to performance outcomes  Motivated and driven to achieve high standards. | Strives to deliver policy and protocol without deviation  Focus much more upon demands of individual patient, then ICU and external demands of hospital.  Unwilling to compromise in terms of staffing ratios, finds shortage of resources very stressful.  Very fixated upon concept of patient and unit safety.  Preoccupied with own responsibility and accountability | "he obviously, in my mind, would need a one to one.'  'we would have to put them on hold for the moment.  It is not safe'.  "I need more people, simple as.  There is no other.  So whether that is the other units helping out, agency coming in, swapping people off shifts, getting the educators in just to give a hand."  " I like to know more in-depth because at the end of the day you are in charge of this shift.  You have to be accountable for it."  "I do understand the pressures from consultants to try to admit and they are looking after a patient, but actually we have to look after what is on the unit safely.  That’s the way I would feel"  "So breaks are going to have to be started very early, people are going to have to be on time if they want adequate breaks, just to maintain numbers"  "not a lot makes me feel very comfortable. It’s all quite challenging, because you’re having to support 12 patients with fewer nurses than is really safe, "  "You absolutely have to prioritise my patient. You have to send me the nurses. I need to wash and roll. Literally. I can’t be any clearer"  "we haven’t even started talking about actually staff safety and staff having breaks and actually being allowed time to get away from the unit and de-stress.  I think that has an impact on patient care.  If someone is feeling overworked and overwhelmed they themselves can become less effective."  " I just think you feel very stressed, you feel very overworked, frustrated that you can’t do everything and you can’t help people and, actually, personally I found that I just wanted people to stop asking me questions and stop asking me things because I can’t process all this information and try to keep up with what’s going on with the patients and to make sure they’re safe."  "then they will try to push to transfer somebody else if they can and then you have not enough staff to look after the patient, and you’re thinking about the safety of the unit."  "safer because you’re looking after a patient and you can concentrate"  "We cannot transfer the patient, even their still booked elective or five, we have to make sure that this patient has got no pain before we transfer, because at least the patient is here in ITU, we can closely monitor them, so just for example, the patient is not ready to go in the ward, during our shift."  "So the unit is full with twelve patients so I would want, safely, twelve nurses to look after the patients"  "would really want to know that actually direct supervision is happening it’s not just… so they are following the right protocols and procedures."  "I feel challenged by the fact that I’ve got a full unit"  "you’ve got more control over what you can do with them if they’re sedated.  It’s these patients that are sort of level 2s but can suddenly deteriorate that I wouldn’t be comfortable with them"  "Barriers are like, just because we’re doubling there is not enough time to spend with each of the patients, so there will be less hands-on time and lots of documentation to look for, for both of them.  So, basically, you don’t give hands-on care for the patient and just go on doing whatever basic needs to be done"  "Yeah, I will have to go through every patient’s plan for the day and make sure as much as possible it gets done by the end of the day.  And it’s hard sometimes, when they are low staffing levels, to achieve everything.  But then, as I said, even when they are doubling they just tend to do what’s important, what’s immediate and generally what needs to be done so that minor details get overlooked.  So then I will have to go and make sure that that’s done" |
